# Antitumor activity of G-quadruplex-interactive agent TMPyP4 with photodynamic therapy in ovarian carcinoma cells

**DOI:** 10.3892/ol.2014.2125

**Published:** 2014-05-08

**Authors:** HONGLI LIU, CHANGSHUAI LV, BAIJUAN DING, JIE WANG, SHAN LI, YOUZHONG ZHANG

**Affiliations:** Department of Obstetrics and Gynecology, Qilu Hospital of Shandong University, Jinan, Shandong 250012, P.R. China

**Keywords:** human ovarian carcinoma, photodynamic therapy, minichromosome maintenance protein-2, carbonic anhydrase IX, 5,10,15,20-tetra-(N-methyl-4-pyridyl) porphine

## Abstract

The aim of the present study was to investigate the potential effects of photodynamic therapy (PDT) mediated by the cationic porphyrin, 5,10,15,20-tetra-(N-methyl-4-pyridyl)porphine (TMPyP_4_), on an ovarian carcinoma cell line and the underlying mechanisms by which TMPyP_4_-PDT exerts its actions. The analysis of cell viability, hematoxylin and eosin staining and flow cytometric apoptosis assays revealed that TMPyP_4_-PDT potently suppressed the growth of the A2780 cells in a laser energy- and dose-dependent manner. Mechanically, it was observed that TMPyP_4_-PDT suppressed the proliferation and motility of the A2780 cells. In addition, the expression levels of minichromosome maintenance protein-2 (MCM2) and carbonic anhydrase IX (CA-IX) were detected by western blot analysis. The results indicated that the TMPyP_4_-PDT-induced apoptosis and antimetastatic activity in the A2780 cells was accompanied by the inhibition of the expression of MCM2 and CA-IX. Therefore, TMPyP_4_-PDT may represent a potential therapeutic method for the treatment of ovarian carcinoma.

## Introduction

As ovarian cancer cells are only superficially invasive and primarily disseminate within the peritoneal cavity, ovarian carcinoma biology differs from that of hematogenously metastasizing tumors. However, as ovarian carcinoma is only temporarily chemosensitive, presents with rapidly proliferating tumors that compress the visceral organs and has a cure rate of only 30%, the disease is lethal (1). Conventional treatments, including surgery, chemotherapy and radiation, are basically ineffective, as surgical procedures are limited by disease staging, chemotherapy may increase the risk of side-effects and radiotherapy may cause serious local tissue injury(2–6). Therefore, the identification of a safe and effective therapy for the treatment of ovarian carcinoma is required.

Photodynamic therapy (PDT) is a novel technology for the treatment of tumors; it is a minimally invasive therapeutic modality that has been demonstrated to be effective in several types of cancer and non-oncological conditions (7). The cationic porphyrin, 5,10,15,20-tetra-(N-methyl-4-pyridyl) porphine (TMPyP_4_), is a novel type of synthetic water-soluble photosensitizer. TMPyP_4_ binds to and stabilizes G-quadruplexes and has been revealed to form G-quadruplexes in the promoter or regulatory regions of important oncogenes, including c-myc, c-myb, c-fos and c-Abl (8), as well as in the single-stranded G-rich overhangs of telomeres *in vitro* (9). Additionally, it has been reported that the nuclear-mitochondrial shuttling of telomerase reverse transcriptase and mitochondrial dysfunction are involved in the arrest of cell proliferation that is induced by the G-quadruplex ligand, TMPyP_4_ (10). These observations indicate that the G-quadruplex structure presents a potential therapeutic target in tumor cells. However, the precise effect of TMPyP_4_-PDT against ovarian carcinoma cells and the underlying molecular mechanisms have not yet been established.

In the current study, the apoptotic effect of TMPyP_4_-PDT on tumor cells and the expression levels of minichromosome maintenance protein-2 (MCM2) and carbonic anhydrase (CA)-IX were investigated by analyzing the apoptotic rate of the human ovarian carcinoma A2780 cell line *in vitro* in order to highlight the clinical significance of TMPyP_4_-PDT in the treatment of ovarian carcinoma patients.

## Materials and methods

### Cell lines and reagents

The human ovarian carcinoma A2780 cell line was obtained from the Cancer Center Laboratory of Shandong University (Jinan, Shandong, China) and cultured in RPMI-1640 (HyClone, Logan, UT, USA) with 10% fetal bovine serum (FBS; HyClone), containing 2 mM L-glutamine, 100 U/ml penicillin and 100 μg/ml streptomycin (Shanghai Solarbio Bioscience and Technology Co., Ltd., Shanghai, China) in an atmosphere of 5% CO_2_ and 100% humidity at 37°C.

TMPyP_4_ was purchased from Calbiochem (San Diego, CA, USA) and was stored with minimal exposure to light. In addition, suspension cultures were exposed to a single laser at the energy densities of 0, 3, 6 and 12 J/cm^2^ by a 800-mW power and 630-nm wavelength semiconductor laser (B100, Zhengzhou Zhongxing Medical Equipment Co., Ltd., Henan, China).

The primary antibodies against anti-human MCM2 (rabbit polyclonal), anti-human CA-IX (rabbit monoclonal) and anti-human GAPDH (rabbit monoclonal), and horseradish peroxidase (HRP)-conjugated goat anti-rabbit and anti-mouse IgG were purchased from Cell Signaling Technology, Inc. (Beverly, MA, USA).

### Cell viability

The cell viability was assessed using the 2-(2-m- ethoxy-4-nitrophenyl)-3-(4-nitrophenyl)-5-(2,4disulfopheny)-2H-tetrazolium, monosodium salt cell counting kit-8 (CCK-8) assay (Bestbio Biotechnology, Shanghai, China). Fresh cells were seeded in 96-well flat-bottomed tissue culture plates (Corning Inc., Corning, NY, USA) at a concentration of 4×10^3^ cells/well with complete culture medium and incubated for 24 h. Following two washes with phosphate-buffered saline (PBS), the cells were incubated in 100 μl culture medium containing 3, 6, 15, 30 or 60 μM TMPyP_4_ for 4 h. Next, cells at each concentration were exposed to the single laser at an energy density of 0, 3, 6 and 12 J/cm^2^, respectively. Following irradiation, the cells were incubated in fresh medium for an additional 24 h at 37°C prior to the CCK-8 assay. A total of 10 μl CCK-8 and 100 μl RPMI-1640 culture medium was then added to each well, and following incubation for 1 h at 37°C, the optical densities of the samples were measured directly using a spectrophotometric microplate reader (Beyotime Institute of Biotechnology, Haimen, China) at a wavelength of 450 nm. Each experiment was performed in triplicate and repeated five times.

### Morphological observations

Following treatment with TMPyP_4_ for 4 h, the cells were incubated for an additional 24 h prior to morphological analysis of the cells observed under an inverted microscope (Olympus, Tokyo, Japan). The laser energy density was set at 6 J/cm^2^ and the cells were stained using a hematoxylin and eosin staining kit (Beyotime Institute of Biotechnology).

### Analysis of apoptotic cells

The apoptotic cells were identified using the Annexin V-fluorescein isothiocyanate apoptosis detection kit (Bestbio Biotechnology). The cells were then incubated for 24 h following irradiation and three washes with PBS. The cell concentration was then adjusted to 5×10^5^ cells/ml. Next, the apoptotic cells were analyzed using FACSCalibur (BD Biosciences, San Jose, CA, USA), and the data was analyzed using FlowJo 7.6.1 software (TreeStar, Inc., Ashland, OR, USA).

### Western blot analysis

Following treatment with 3, 6 or 15 μM TMPyP_4_ for 4 h and laser treatment, the cells were incubated for an additional 48 h prior to the collection cells for protein extraction. The examination of the expression levels of MCM2 and CA-IX was then performed separately. The laser energy density was set at 6 J/cm^2^ and total protein was extracted with the radioimmunoprecipitation assay reagent in the presence of phosphatase protease inhibitors (Beyotime Institute of Biotechnology) and the bicinchoninic acid assay kit (Beyotime Institute of Biotechnology) was used to measure the protein concentration. The protein (50 μg) was separated by SDS-PAGE and transferred onto a polyvinylidene fluoride membrane using wet transfer apparatus (Bio-Rad, Hercules, CA, USA). The membranes were then blocked with 5% skimmed milk and incubated overnight at 4°C with the primary antibodies, followed by incubation with the secondary antibodies labeled with HRP. Next, the protein bands were visualized using an enhanced chemiluminescence kit (Millipore, Billerica, MA, USA) and the protein levels were detected using the chemiluminescence reader, ImageQuant™ LAS4000 (GE Healthcare, Pittsburgh, PA, USA), and analyzed by ImageJ software (US National Institutes of Health, Bethesda, MD, USA).

### Statistical analysis

Data are presented as the mean ± standard deviation. Student’s two-tailed t-test was used to determine the statistical differences between the treatment and control groups. P<0.05 was considered to indicate a statistically significant difference.

## Results

### Effects of TMPyP_4_-PDT on cell growth

**Figure 1:**
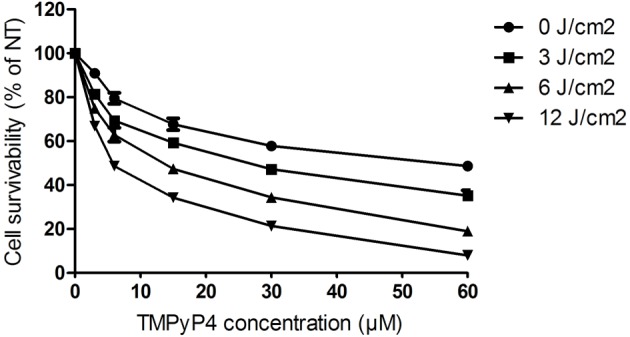
Effects of TMPyP_4_-PDT on the cell growth of A2780 cells. In the TMPyP_4_-PDT group, the cells were incubated with 3, 6, 15, 30 or 60 μM TMPyP_4_ and then exposed to single laser energy at densities of 0, 3, 6 and 12 J/cm^2^ with a semiconductor laser. TMPyP_4_, 5,10,15,20-tetra-(N-methyl-4-pyridyl)porphine; PDT, photodynamic therapy.

The results showed that treatment with PDT alone at various laser energy densities did not significantly inhibit the growth of the A2780 cells (P>0.05). However, treatment with TMPyP_4_ alone at doses of 3, 6, 15, 30 or 60 μM significantly inhibited the growth of the cells (P<0.05) in a dose-dependent manner. Furthermore, treatment with TMPyP_4_-PDT at doses of 3, 6, 15, 30 or 60 μM also significantly inhibited the growth of the cells, and these inhibitory effects were observed to be in a laser energy- and dose-dependent manner (P<0.01 and P<0.01, respectively) (Fig. 1).

### Effects of TMPyP_4_-PDT on cell morphology

**Figure 2:**
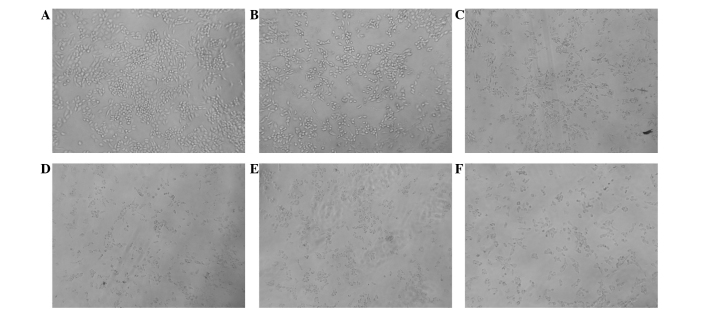
TMPyP_4_-PDT induces various morphological changes in A2780 cells. Representative images of A2780 cells treated with TMPyP_4_-PDT captured by inverted microscopy are shown (magnification, ×200). (A) Blank control group and groups treated with TMPyP_4_-PDT concentrations of (B) 3, (C) 6, (D) 15, (E) 30 and (F) 60 μM were exposed to a single laser energy density of 6 J/cm^2^. TMPyP_4_, 5,10,15,20-tetra-(N-methyl-4-pyridyl)porphine; PDT, photodynamic therapy.

The cells in the blank control were observed to be polygonal or spindle-shaped. The cells treated with 3 μM TMPyP_4_ were partially round in shape, whereas cells treated with 6 μM TMPyP_4_ were in a poor state and adherent cells were sparse. Furthermore, with increasing TMPyP_4_ concentration, the cells became increasingly round in shape and the number of adherent cells was significantly reduced. In addition, apoptotic bodies were clearly visible following treatment with 6 μM TMPyP_4_ (Fig. 2).

**Figure 3:**
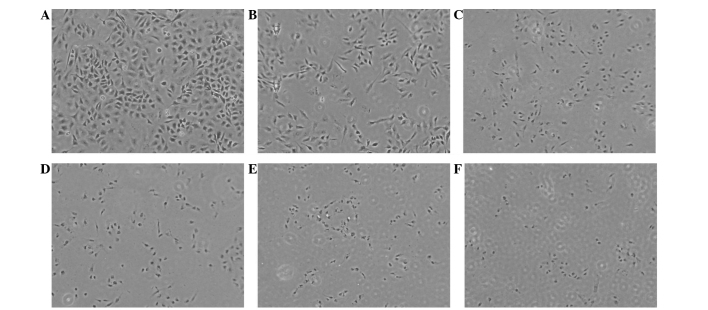
TMPyP_4_-PDT induces various morphological changes in A2780 cells. Representative hematoxylin and eosin staining images of A2780 cells captured by inverted microscopy are shown (magnification, ×200). (A) Blank control group and groups treated with TMPyP_4_-PDT concentrations of (B) 3, (C) 6, (D) 15, (E) 30 and (F) 60 μM were exposed to a single laser energy density of 6 J/cm^2^. TMPyP_4_, 5,10,15,20-tetra-(N-methyl-4-pyridyl)porphine; PDT, photodynamic therapy.

Under the conditions of the laser energy density set at 6 J/cm^2^, the blank control group morphology was more consistent with a high nuclear cytoplasm ratio. Furthermore, with increasing TMPyP_4_-PDT concentration, the cell morphology of the TMPyP_4_-PDT group became increasingly irregular and nuclear pyknosis or fragments were observed (Fig. 3).

### Effects of TMPyP_4_-PDT on cell apoptosis

**Figure 4:**
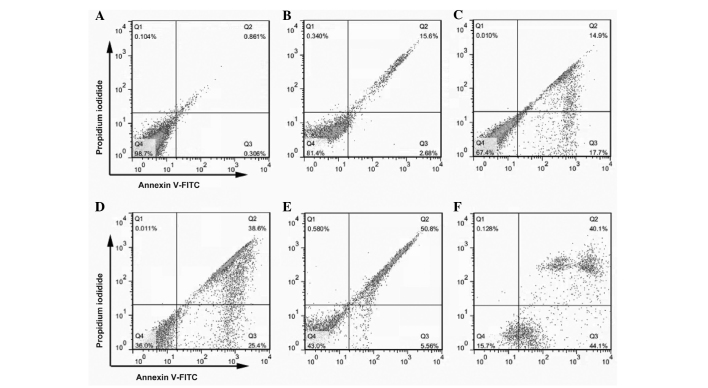
TMPyP_4_-PDT induces cell apoptosis in A2780 cells. The cells were cultured with 3, 6, 15, 30 or 60 μM TMPyP_4_ for 4 h prior to exposure to radiation and incubation at 37°C for 24 h. The apoptotic cells were identified using the fluorescent marker, Annexin V-fluorescein isothiocyanate (magnification, ×200). (A) Blank control group and groups treated with TMPyP_4_-PDT concentrations of (B) 3, (C) 6, (D) 15, (E) 30 and (F) 60 μM were exposed to a single laser energy density of 6 J/cm^2^. TMPyP_4_, 5,10,15,20-tetra-(N-methyl-4-pyridyl)porphine; PDT, photodynamic therapy.

Propidium iodide and Annexin V double-staining were used to detect the changes in apoptotic cells treated with TMPyP_4_-PDT, and the apoptosis-inducing effects of TMPyP_4_-PDT were assessed using FlowJo software. Under the conditions of the laser energy density set at 6 J/cm^2^, the effects of TMPyP_4_-PDT on the apoptosis of the A2780 cells was examined following treatment at doses of 3, 6, 15, 30, or 60 μM. The cells that were treated with the various doses of TMPyP_4_ for 24 h showed a significant increase in the number of apoptotic bodies compared with the negative control group, and the apoptotic cell percentage increased in a dose-dependent manner. The cell apoptosis rates were 1.0±0.10, 14.7±2.22, 32.3±1.69, 52.2±1.47, 56.3+1.23 and 80.3±3.14% for doses of 3, 6, 15, 30, or 60 μM TMPyP_4_, respectively (Fig. 4).

### Effects of TMPyP_4_-PDT on the expression levels of MCM2 and CA-IX proteins

**Figure 5:**
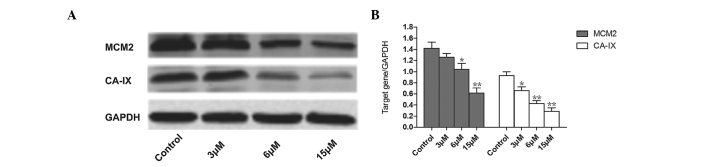
TMPyP_4_-PDT reduces the levels of MCM2 and CA-IX expression in A2780 cells. (A) Western blot analysis detected the levels of MCM2 and CA-IX expression in the A2780 cells, and GAPDH was used as a control. (B) Statistical graph of MCM2 and CA-IX expression. Significant differences in expression were identified between the groups for the two genes (P<0.05). Data are presented as the mean ± standard deviation of three independent experiments. ^*^P<0.05 and ^**^P<0.01 vs. control group. TMPyP_4_, 5,10,15,20-tetra-(N-methyl-4-pyridyl)porphine; PDT, photodynamic therapy; MCM2, minichromosome maintenance protein-2; CA-IX, carbonic anhydrase-IX.

Following irradiation with a laser energy density of 6 J/cm^2^ and treatment with TMPyP_4_-PDT at doses of 3, 6 or 15 μM, the MCM2 and CA-IX expression levels were analyzed. In contrast to the increased apoptosis observed in A2780 cells, the expression levels of MCM2 and CA-IX were significantly downregulated in a dose-dependent manner (Fig. 5).

## Discussion

PDT is minimally invasive, with good tolerance, improved aesthetic outcomes, minimal functional disturbance, low morbidity and the ability to be used more than once at the same site in comparison to surgery, radiation and chemotherapy. The main mechanism of PDT used for the diagnosis and treatment of cancer involves the use of sensitized molecular components, which transfer heat to generate singlet oxygen through a series of photochemical and photobiological reactions. Furthermore, the generated singlet oxygen and the release of prostaglandins, lymphokines, thromboxane or other cytokines destroys the tumor microvessel and biofilm, thereby killing the tumor cells (11–14). TMPyP_4_ is a tetravalent cationic water-soluble porphyrin that accumulates in tumors, with a good degree of selectivity (15,16). TMPyP_4_ connects with telomeres and promotes telomerase ends to form a stable G-quadruplex structure, which reduces the telomerase activity and its role in tumor cells, and leads to the arrest of tumor cell growth, thus inducing the apoptosis of tumor cells (17,18). In the current study, the growth inhibition of the human ovarian carcinoma A2780 cells was not evident in the PDT group (P>0.05). However, in the TMPyP4 and TMPyP4 PDT groups, the growth inhibition of the human ovarian carcinoma A2780 cells gradually increased with increasing drug concentration and laser energy density, although, when the treatment reached a certain level, the inhibition of the cell increase slowed down (P<0.05). Therefore, in clinical PDT, an appropriate dose of the photosensitizer and laser energy density must be selected, as blindly increasing the dose of the photosensitizer and laser energy density is not conducive to improving the efficacy and may lead to toxicity and side-effects as a result of the high dose. The effect of 5-aminolevulinic acid-mediated PDT on the induction of apoptosis has also been extensively described (19,20). Consistent with previous studies, a similar phenomenon was observed in the current study, whereby the TMPyP_4_-PDT-induced cell apoptosis and the effect on the induction of apoptosis were in a laser energy- and dose-dependent manner (21).

MCM2 is one of the conserved set of six related proteins of the MCM complex (MCM2–7), which is essential in the regulation of DNA replication (22). MCM2 has been studied in a wide range of human organs, and its overexpression has been identified in various types of tumors and tumor-like lesions of the oral mucosa, larynx, stomach, colon, esophagus, breasts, lungs, ovaries, kidneys, prostate, bladder, brain and soft tissues (23,24). CA-IX is a member of the CA family, which are a group of zinc-containing metalloenzymes that catalyze the reversible hydration of carbon dioxide to carbonic acid and are involved in respiration and the acid-base balance (25). CA-IX is important in the regulation of cell proliferation and transformation, and is conducive to tumor growth and metastasis (26). The present study demonstrated that the antitumor effects of TMPyP_4_-PDT are accompanied by the downregulation of MCM2 and CA-IX. According to this result, we hypothesized that TMPyP_4_-PDT may inhibit the DNA replication of tumor cells and change pH homeostasis, thereby inhibiting tumor cell proliferation and metastasis. However, the specific mechanism of TMPyP_4_-PDT requires further study.

In conclusion, the present study demonstrated that TMPyP_4_-PDT potently suppressed the cell growth of the A2780 cells in a laser energy- and dose-dependent manner. In addition, the study indicated that TMPyP_4_-PDT may exhibit its antitumor activity by downregulating the expression of MCM2 and CA-IX in human ovarian carcinoma cells. Furthermore, TMPyP_4_ enhances laser sensitivity. These results indicated that TMPyP_4_-PDT may be supplementary to conventional therapy in the treatment of ovarian carcinoma. Further study to clarify the molecular mechanism of the TMPyP_4_-PDT-induced antitumor activity may provide a rationale for the development of antitumor drug targeted therapies for ovarian carcinoma.
